# Genome-Wide Identification of GmPIF Family and Regulatory Pathway Analysis of GmPIF3g in Different Temperature Environments

**DOI:** 10.3390/ijms26020551

**Published:** 2025-01-10

**Authors:** Xuefeng Liang, Caitong Zhao, Jiayang Cui, Zhihua Liu, Dezhi Han, Qingshan Chen, Mingliang Yang, Zhenfeng Jiang

**Affiliations:** 1 National Key Laboratory of Smart Farm Technologies and Systems, Northeast Agricultural University, Harbin 150030, China; liangxf1230@163.com (X.L.); m18846773398@163.com (C.Z.); 18345555714@163.com (J.C.); qshchen@126.com (Q.C.); 2 College of Resources and Environment, Northeast Agricultural University, Harbin 150030, China; zhihua-liu@neau.edu.cn; 3 Heihe Branch of Heilongjiang Academy of Agricultural Sciences, Heihe 164300, China; handezhi2008@163.com

**Keywords:** soybean, PIF, bioinformatics analysis, biofilm interference, interacting protein

## Abstract

Phytochrome-interacting factors (PIFs) play a crucial role in regulating plant growth and development. However, studies on soybean PIFs are limited. Here, we identified 22 GmPIF genes from the soybean genome and classified the GmPIF proteins into 13 subfamilies based on amino acid sequence homology, secondary and tertiary structures, protein structure, and conserved motifs. Genome-wide collinearity analysis revealed that fragment duplication events play a dominant role in expanding the GmPIF gene family. Cis-acting element analysis revealed that the GmPIF gene family is involved in light response, hormone response, biotic–abiotic stress response elements, and plant growth and development. Gene expression analysis in different temperature environments showed that the GmPIF family was found to be induced by phytohormone treatments, with a significant increase in the expression level of *GmPIF3g*. *GmPIF3g* plays a key role in the regulation of the entire network, and in addition, 30 proteins interacting with the *GmPIF3g* promoter were identified through the use of a novel biofilm interference technique. This technique showed that the transcription factor Dof (DNA binding with one finger) binds to the *GmPIF3g* promoter, and Y1H assays indicated that Dof regulates its expression by binding to the PIF promoter. These results provide a theoretical basis for further studies on the regulatory network of GmPIF genes to improve the structure of soybean plants under shade environments, as well as a new method for analyzing regulatory elements that interact with gene promoters.

## 1. Introduction

Phytochrome-interacting factors (PIFs) are crucial plant photoreceptors that belong to the bHLH (basic-helix-loop-helix) transcription factor family. In *Arabidopsis thaliana*, eight PIF members have been identified, including PIF1, PIF3, PIF4, PIF5, PIF6, PIF7, and PIF8 [1,2,3,4,5,6]. PIFs have a conserved APB (active phytochrome B-binding motif) or APA (active phytochrome A-binding motif) at the N-terminal end and a conserved bHLH structural domain at the C-terminal end. PIF3 was the first PIF member identified; yeast two-hybrid technology revealed that it interacts with the C-terminal end of PhyB (Phytochrome B) [4]. Pr will change into Pfr (far-red light absorption form) when exposed to red light and move from the cytoplasm to the nucleus. It binds to PIF’s APB or APA domains, causing the PIF to switch from an inhibitory to an active state. Activated PIF may bind to particular DNA sequences, control downstream gene expression, and influence plant growth and development [7]. All PIFs are particularly connected to PhyB via Pfr. Arabidopsis phyB, once activated by light, may engage directly with a kind of phytochrome interaction factor PIF, transferring light signals and influencing downstream gene expression, so boosting photomorphogenesis. As a result, phyB and PIFs are critical signal modules that allow plants to respond to their surroundings in light. Recent research has shown the exact molecular process of light-activated phyB from Pr to Pfr, proposing the “induction-fit” interaction model between phyB and PIF [8]. PIF1 and PIF3 both contain an APA domain, which can interact with PHYA (phytochrome A). However, this interaction is not conserved structurally. According to certain research, PIF1 binds more strongly with PHIA than PIF3. In this technique, the PIF gene is coupled with the photo-responsive gene to control their expression levels in Arabidopsis by increasing PIF phosphorylation [9].

The PIF serves as a central regulator of intracellular signaling, participating in the modulation of numerous biological processes in plants. It not only controls plant growth and development but also plays a crucial role in the plant’s resistance mechanisms by integrating various plant hormone signaling pathways. During the plant’s response to abiotic stresses such as low temperature, high temperature, shading, and drought, PIF exerts a pivotal regulatory function [9,10,11,12,13,14,15]. In the process of plant growth and development, different hormone pathway signals are also related to PIF, especially those involving hormones: GA (gibberellin) [16,17,18], ABA (abscisic acid) [19,20], BR (brassinosteroid) [21], JA (jasmonate) [22], IAA (auxin) [23,24], and ethylene [25]. GA and BR participate in the signal transduction of light and temperature. DELLA (Asp Glu Leu Leu Ala) is a negative regulator of the GA pathway that responds to light and circadian rhythms by regulating PIF activity. At night, GA and GID1 (GA-insensitive dwarf 1) concentrations reach their highest levels, which inhibit the activity of DELLA to negatively regulate PIF. Increased PIF mRNA levels subsequently promote rapid hypocotyl growth before dawn [26]. At dawn, the decrease in GID1 levels leads to the stabilization of DELLA and the suppression of PIF activity, which subsequently results in chlorophyllation and a reduction in plant growth [27]. During the daytime Pfr phosphorylates PIFs, which, in turn, degrades PIFs via the ubiquitin–proteasome pathway. An insufficient amount of PIFs inhibits hypocotyl growth [28,29].

There is extensive overlap between the genes regulated downstream of BZR1 and those related to light signaling [30]. PIF4 and active BZR1 build up and interact with one another under dark conditions to encourage hypocotyl elongation [31]. Conversely, pifq mutants are less sensitive to exogenous oleuropein lactones than the wild type under light conditions; in the bri 1–116 mutant, the overexpression of the non-functional BZR1 results in a dwarfing phenotype due to the upregulation of PIF4 [32]. BIN2, as a negative regulator of the BR signaling pathway, regulates periodic hypocotyl growth by phosphorylating PIF4 [33]. PIF activates BR signaling kinase 5, promoting the interaction between the downstream transcription factor BES1 and the PIF, forming a BES1-PIF complex to enhance downstream gene expression [34]. In addition, PIF4 also functions in controlling hypocotyl elongation in Arabidopsis by regulating the IAA concentration. In warm environments, PIF4 accumulates as it interacts with the transcriptional activator HMR to promote the expression of heat-sensitive growth-related genes–YUC8, IAA19, and IAA29–thereby stimulating plant growth [35]. Moreover, PIF4 interacts with the transcriptional cofactor SEU to form a regulatory complex that regulates the expression of the early hormone response genes IAA6 and IAA19 and affects IAA concentration to promote hypocotyl elongation [36]. Under shade conditions, the enhanced activity of PIF4, PIF5, and PIF7 promotes the expression of YUC8, IAA19, and PRE1 genes, thereby promoting plant elongation [37].

As a fixed plant, soybean must integrate a variety of external and endogenous cues to regulate its height, and the internode length of soybean plants is critical in determining plant height and overall structure. Exogenous and endogenous hormones, as well as light and temperature, stimulate internode elongation. Plant hormones control internode elongation via concentration fluctuations and steady-state changes [38]. Light and temperature control internode elongation by coordinating the light-temperature signal route with the plant hormone system [6]. The mechanism of PIF-mediated light and temperature control, as well as the integration of the hormone signal route, remain unknown.

In this work, we sought to uncover and describe the integrated regulatory mechanism of soybean PIF gene family members in light, temperature, and hormone signaling pathways. In order to do this, we examined their expression patterns in various light, temperature, and hormone settings. We employed a novel method called biofilm interference (BLI) to discover proteins that interact with the critical factor *GmPIF3g* promoter. Y1H analysis was utilized to confirm the combination of Dof and PIF promoters. These findings lay a good framework for further research into the involvement of the *GmPIF* gene in the light, temperature, and hormone control network, which may improve the structure of soybean plants grown in shady environments. In addition, in this study, we proposed a new method to fish the binding protein with the gene promoter.

## 2. Results

### 2.1. Identification of GmPIF Genes in Soybean

By analyzing soybean transcriptome data, a total of 22 candidate PIF protein sequences were obtained and named GmPIF1a-GPIF1e, GmPIF3a-GmPIF3k, GmPIF4a-GmPIF4d, GmPIF7a, and GmPIF7b. The basic physicochemical properties of GmPIF protein sequences were analyzed using ExPaSy. The molecular weight range of the 22 members of the GmPIF gene family is 18.48–77.25 kD, encoding amino acids ranging from 160 to 633 aa, with a maximum isoelectric point of 9.41 (GmPIF7b) and a minimum of 5.61 (GmPIF3a). Among them, GmPIF1d, GmPIF3b, GmPIF3d, GmPIF3f, GmPIF3h, GmPIF3j, and GmPIF7b have a PI > 7, indicating alkalinity, while others have a PI < 7, indicating acidity. Only two GmPIF proteins are stable (coefficient less than 40), indicating poor stability of the GmPIF transcription factors. In total, 22 GmPIF members are distributed on 13 chromosomes and located in the nucleus (Table 1), where they perform the most important biological functions.

### 2.2. Phylogenetic Tree Construction, Motif, and Gene Structure Analysis

**Figure 1:**
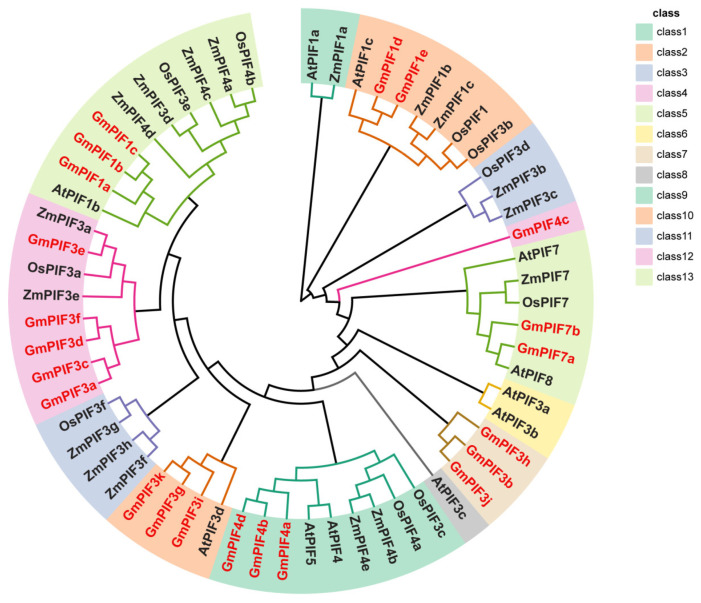
Phylogenetic analysis of the GmPIF family. Note: Phylogenetic analysis of phytochrome-interacting factor (PIF) family members from rice (Os), maize (Zm), Arabidopsis (At), and soybean (Gm). Color blocks pertaining to the different PIF classes are shown on the right;the red color in the picture highlights the GmPIF family members.

Based on the phylogenetic tree, which is constructed to analyze the sequence homology and explore the evolutionary affinities of PIFs among different species as well as within the members of the GmPIF family. By comparing the full-length protein sequences of 22 PIFs from soybean, 17 PIFs from maize, 11 PIFs from rice, and 11 PIFs from Arabidopsis, all the proteins were categorized into 13 distinct subfamilies, ranging from class 1 to 13 (Figure 1). There were no homologous genes in classes 1, 3, 6, 8, and 11. The homology of GmPIF family members with Arabidopsis was significantly greater than that of monocotyledonous plants such as rice and maize. The high homology observed between the members of the GmPIF family and the members of the *AtPIF* family indicates the presence of similar biological functions. GmPIF1d and GmPIF1c were clustered in class 2 and were homologous to AtPIF1c. GmPIF4c was clustered in class 4 and was more distantly related to the other genes. GmPIF7a and GmPIF7b were clustered in class 5 and were homologous to AtPIF7 and AtPIF8. GmPIF3h, GmPIF3b, and GmPIF3j were clustered in class 7. GmPIF4a, GmPIF4b, and GmPIF4d were homologous to AtPIF4 and AtPIF5. GmPIF3k, GmPIF3g, and GmPIF3i were homologous to AtPIF3d. GmPIF3a, GmPIF3c, GmPIF3d, GmPIF3f, and GmPIF3e were clustered in class 12. GmPIF1a, GmPIF1b, and GmPIF1c were homologous to AtPIF1b and were clustered in class 13.

**Figure 2:**
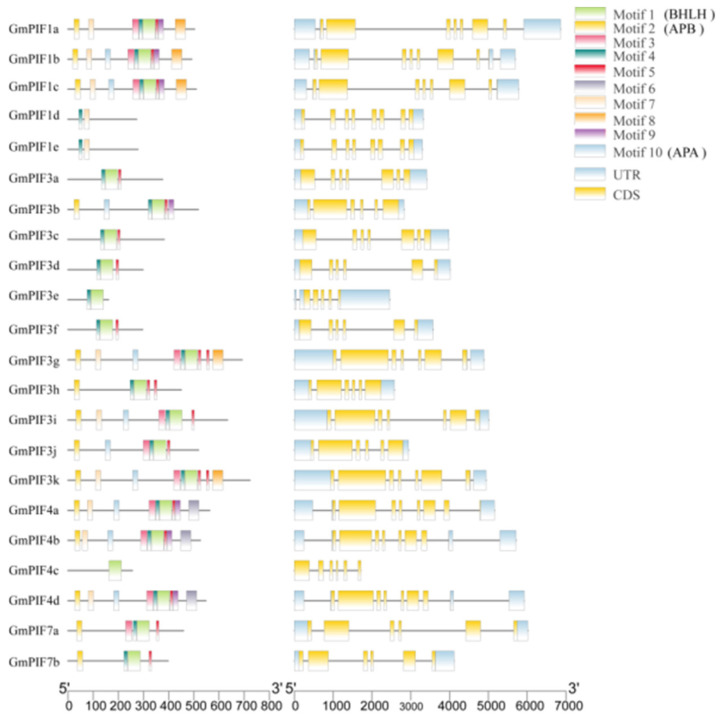
The conserved protein sequence motif of GmPIF family and the gene structure of *GmPIF* family. Note: protein sequence motifs of members of GmPIF family and gene structure of *GmPIF* family. Color blocks are displayed on the right.

In proteins encoded by genes belonging to the GmPIF family, 10 motifs were found (Figure 2). (Supplemental Table S1), and all GmPIFs contained Motif1 (BHLH motif) except GmPIF1d and GmPIF1e. Except for GmPIF1d, GmPIF1e, GmPIF3a, GmPIF3c, GmPIF3d, GmPIF3f, and GmPIF4c, the other GmPIFs contained Motif2 (APB motif), which is the most important motif; all of them exhibited similar exon–intron distribution patterns. The 22 *GmPIF* genes included the UTR and CDS. The number of CDS in each gene varied from five (*GmPIF3e*) to eight (*GmPIF1d* and *GmPIF1e*). The quantity of exons and introns is a crucial marker of the gene family’s evolutionary history, and the makeup of these segments greatly influences a gene’s function. The same type of *GmPIF* genes generally have a similar number of exons but varying numbers of introns, indicating that intron insertion is a key factor in altering gene structure and may become a key factor in regulating its functional differentiation [39].

### 2.3. Predicting the Protein Secondary and Tertiary Structure of the GmPIF Gene

**Figure 3:**
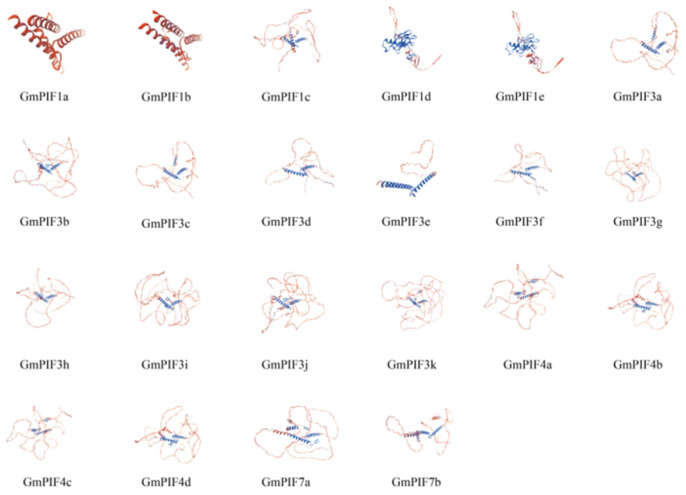
Tertiary structure of the GmPIF family members.

The secondary structure prediction revealed that the α-helix content in the proteins encoded by the PIF gene exceeds 0.15. This finding suggests that the secondary structural characteristics of the proteins encoded by the PIF gene are characterized by a relatively high proportion of α-helices. The gene with the largest β-sheet was GmPIF1e, and the gene with the smallest β-sheet was GmPIF7a (Table 2). Secondary structure analysis revealed that the proportion of irregular curls was the highest, followed by α-helix, while extended strands and β-sheet were the lowest. The α-helix was the most stable of all the protein secondary structures. The lower proportion of α-helix in GmPIF indicates lower stability, which is consistent with the higher instability coefficient observed in the physicochemical analysis (Figure 3). The following pairs exhibited similar tertiary structures: GmPIF3a and GmPIF3c, GmPIF4a and GmPIF4c, and GmPIF1d and GmPIF1e. GmPIF1d and GmPIF1e did not have BHLH structural domains, which is consistent with previous results (Figure 2).

### 2.4. Collinearity Relation of GmPIF

**Figure 4:**
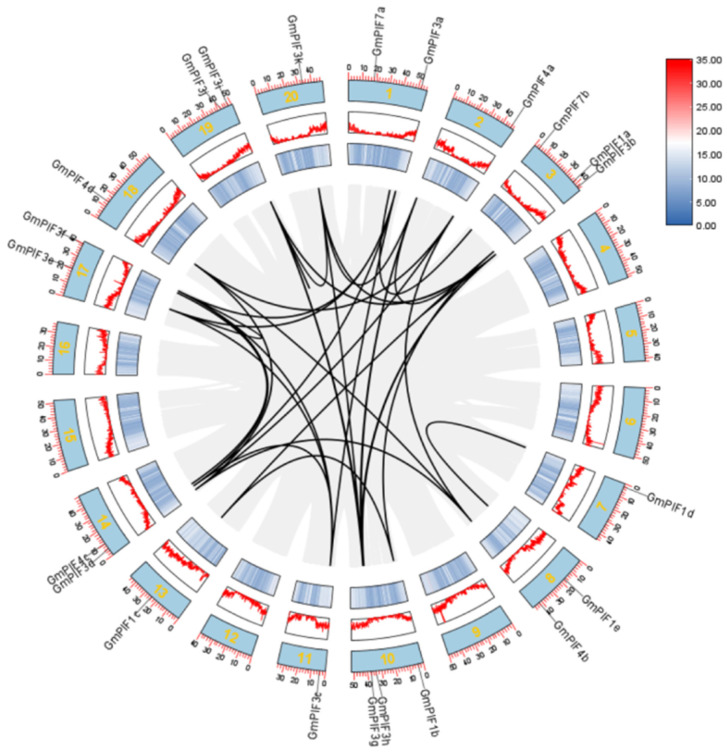
Collinearity analysis of genes in the *GmPIF* family. Note: Homology analysis of 22 *GmPIF* genes. Chromosomes 1–20 are indicated by blue rectangles. Lines, heat maps, and histograms along the rectangles indicate the gene densities on the chromosomes. The red scale shows chromosome lengths in Mb, black highlighted curves show regions of *GmPIF* covariance, and gray curves show genome-wide covariance in soybeans.

To further elucidate the potential functions of the GmPIFs, their intraspecific homology was analyzed (Figure 4). The 22 GmPIFs were distributed on 13 of the 20 soybean chromosomes, each consisting of one to two GmPIFs. Forty homologous gene pairs of GmPIFs were identified (Supplemental Table S2), of which one or more GmPIF genes existed in homologous gene pairs, with the greatest quantity of GmPIF homologous gene pairs on chromosome 10. The findings indicate that fragment replication events were the primary driver behind the expansion of the GmPIF gene family.

### 2.5. Interaction Network Analysis of GmPIF Proteins

**Figure 5:**
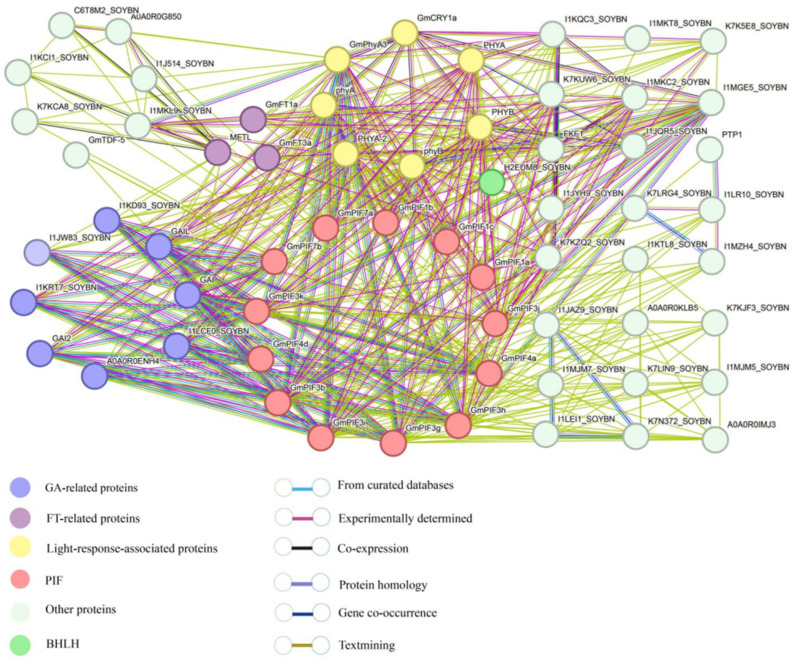
String analysis of the GmPIF family member. Note: Functional interaction networks of GmPIFs in soybean according to orthologs in Arabidopsis. Network nodes represent proteins and lines represent protein–protein associations.

Protein interaction pairs with a composite score greater than 0.4 (medium) were screened from the STRING website, and interaction network analysis revealed the existence of 456 sets of protein interactions (Figure 5). Most of the proteins interacting with the PIF gene family belong to the photosensitive pigments (PHYB, PHYA, GmCRY1a, and I1MGE5) as well as proteins regulating hormone signaling, such as the GA signaling components (GAI, GAI2, and GAI-Like), and the related proteins of the signaling pathway GRAS (A0A0R0ENH4, I1LCF0, I1KD93, I1JW83, and I1KRT7). GmPIF1a, GmPIF1b, and GmPIF1c are involved in the light signaling pathway. GmPIF3a, GmPIF3j, and GmCRY1a have a cooperative role in blue light-induced signal pathway. GmPIF3i, GmPIF3g, GmPIF3k, GmPIF3b, and GmPIF3h have protein interactions with E3 ubiquitin ligase (A0A0R0IMJ3). GmPIF3b, GmPIF3h, and GmPIF3j exist in a regulatory network with FKF1 during cellulose synthesis. GmPIF3j and GmPIF4a interact with FT proteins involved in flower morphogenesis, and GmPIF7a is shown to interact with photochromes in the network interactions diagram; however, the proteins which GmPIF7b interacts with are hormone-associated regulatory factors that do not interact with photochromes. In summary, GmPIFs are widely involved in the complex processes of multiple regulatory networks through interactions with different functional proteins and related transcription factors.

### 2.6. Expression Analysis of GmPIF Family Genes in Different Temperature Environments

**Figure 6:**
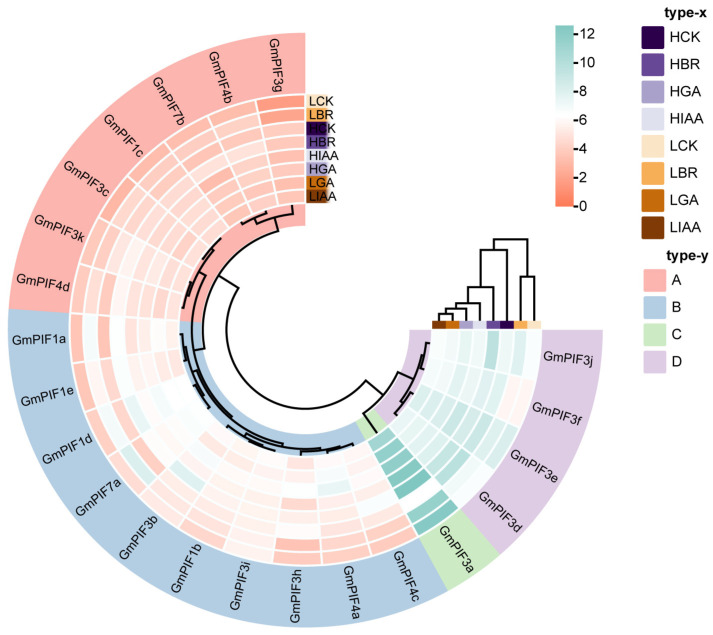
Expression profiles of *GmPIF* under different growth regulator treatments. Note: HCK: High-temperature environment blank control; HBR: BR treatment in high-temperature environment; HGA: GA treatment in high-temperature environment; HIAA: IAA treatment in high-temperature environment; LCK: low-temperature environment blank control; LBR: BR treatment in low-temperature environment; LGA: GA treatment in low-temperature environment; LIAA: IAA treatment in low-temperature environment. The results are shown as a heat map, with red indicating high levels of transcription and green indicating low levels of transcription. The icons are shown on the right side, and the outermost color block is the clustering result.

The levels of *GmPIF3h* and *GmPIF3f* expression varied significantly between HCK and LCK (Figure 6). Furthermore, higher expression levels of *GmPIF3f* were observed in the HCK and HBR groups, whereas lower expression levels were observed in the other six groups. In addition, *GmPIF1a*, *GmPIF1d*, and *GmPIF7a* were downregulated in the HBR and LBR groups. Notably, *GmPIF1d* and *GmPIF7a* showed higher expression levels in the HCK and LCK groups, whereas their expression was significantly downregulated in the other six hormone-treated groups. *GmPIF3b*, *GmPIF4c*, and *GmPIF3f* were downregulated only in the HBR group. Furthermore, under both high- and low-temperature conditions, the expression of GmPIF1d, *GmPIF4a*, and *GmPIF7a* was downregulated after GA treatment. In contrast, *GmPIF3g*, *GmPIF1c*, *GmPIF3c*, *GmPIF4b*, *GmPIF7b*, *GmPIF3k*, and *GmPIF4d* were significantly expressed in all three hormone treatments and control groups. Remarkably, the expression level of GmPIF3g significantly increased, highlighting the more critical role of *GmPIF3g* in the transcriptional regulation of hormone response and temperature regulation.

The response of the PIF genes to different environmental conditions was evident from the observed variations in their expression levels with changes in temperature and hormone treatment. These results suggest that the *GmPIF* gene family, similar to its function in Arabidopsis [40], has a significant part in responding to temperature fluctuations. Additionally, the expression patterns of PIF genes indicate that they are regulated by hormones.

### 2.7. Cis-Acting Element Analysis of the GmPIF Promoter

**Figure 7:**
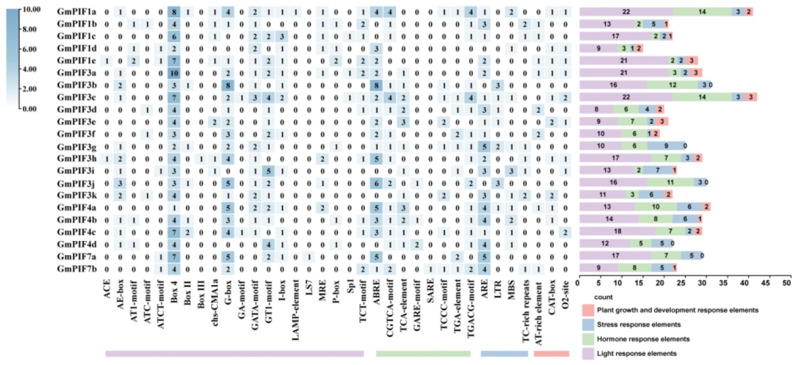
Analysis of cis-acting elements in the promoter region 2 kb upstream of the *GmPIF* start codon. Note: Number of cis-acting elements in the promoter region 2 kb upstream of the translation start site) of the *GmPIF* gene. Right stacking diagram of *GmPIF*. The cis-acting elements were classified into four major categories based on their functional annotations: light, phytohormone responses, abiotic and biotic stress responses, and plant growth and development.

Through the analysis of the homologous elements of the *GmPIF* gene family promoters, the *GmPIF* promoter is classified into four categories: light, hormone, stress, and growth-related elements (Figure 7). There were twenty light-responsive elements, including BOX–4 (ATTAAT); seven hormone-responsive elements; five stress-responsive elements; and three cis-acting elements related to plant growth and development. *GmPIF3* had the highest number (22) of photo-responsive elements. Meanwhile, *GmPIF1a* and *GmPIF3c* had the highest number (14) of hormone-responsive elements, followed by *GmPIF3b*, with 12. *GmPIF3g* contained nine abiotic stress response elements. In total, 15, 17, 12, 11, and 8 genes were identified in MeJA reactivity (CGTCA and TGACG motifs), ABA reactivity, SA reactivity, GA reactivity (GARE and TCCC motifs), and growth hormone reactivity (TGA element). The promoter regions of *GmPIF3c* and *GmPIF3g* contain hormone responsive elements, such as MeJA, ABA, GA, IAA, and SA. The presence of these components reveals the potential regulatory roles of *GmPIF3c* and *GmPIF3g* in plant hormone signaling. The *GmPIF* promoter also contained a number of elements linked to plant growth and development, including cis-elements related to maize alcohol-soluble protein metabolism (O2–site), meristem expression (CAT–box), and AT-rich DNA binding protein (AT-rich element)-related cis-elements.

### 2.8. Identification of GmPIF3g Promoter-Interacting Protein

**Figure 8:**
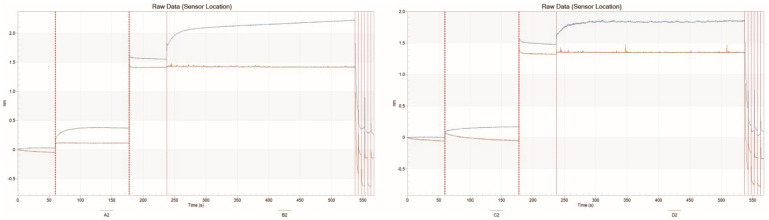
Fishing for *GmPIF3g*−interacting protein using the Octet RED96. Note: A2 is the graph of the binding intensity of *GmPIF3g1* to the inter−combinant protein, and B2 is a graph of the binding intensity of *GmPIF3g1* to the inter−combinant protein solvent. C2 is the binding intensity graph of *GmPIF3g1* with the interacting protein, and D2 is the binding intensity graph of *GmPIF3g1* with the interacting protein solvent.

**Figure 9:**
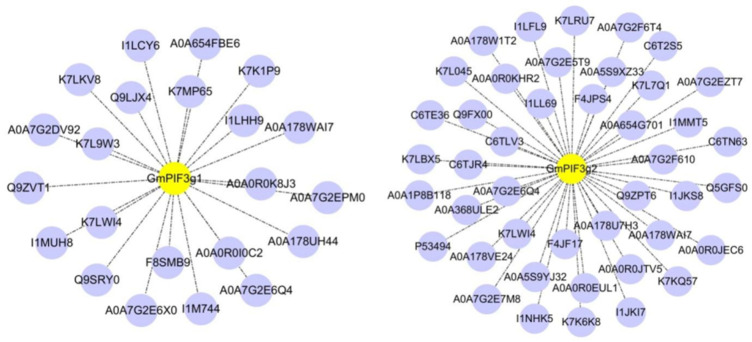
Fishing proteins that bind to the *GmPIF3g* promoter. Note: Yellow circles represent promoters, and purple circles represent proteins bound to promoters.

It is essential to further analyze the transcription factors that bind to the cis-acting elements of the *GmPIF3g* promoter. By designing two promoter probes with a length of 100 bp, we aim to identify the proteins that interact with these probes and further elucidate the interaction network of *GmPIF3g*. In this study, a new method for identifying promoter-interacting proteins using biofilm interference technology (BLI) was selected(Figure 8). A comprehensive functional annotation was conducted on 67 identified interacting proteins by searching in the Uniprot database(Figure 9). The 30 major functional proteins were screened that are mainly involved in the following biological processes (Table 3) including cell wall synthesis, growth hormone synthesis, mitochondria, ubiquitin, DNA-binding family protein, signal transduction, and other functions; pendant protein results match predictions from protein interaction networks. The identified protein functioned varied and suggests there are several regulatory factors that interacted with the *GmPIF3g* promoter. Using the Cell mPLOC database to predict the subcellular localization of the thirty proteins under study, seven were predicted to be located in the nucleus (Table 3).

### 2.9. Y1H

**Figure 10:**
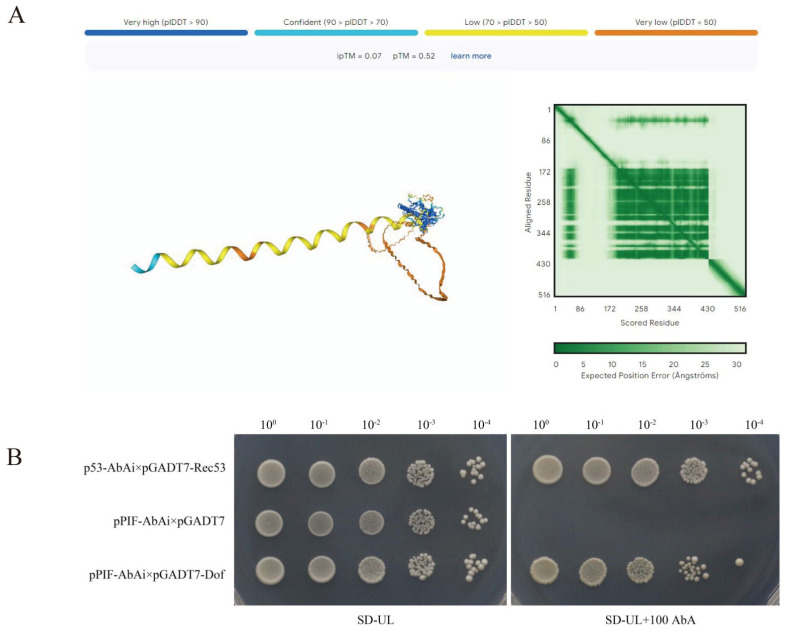
(**A**) Prediction of Interaction between PIF and Dof; (**B**) Yeast monoculture of PIF and Dof.

We use AIPhaFoId3 to predict the possibility of interaction between *GmPIF3g* and Dof, and the results show that the pTM + ipTM score is higher than 0.5, which shows that there is interaction between Gm*PIF3g* and Dof (Figure 10A). The binding of Dof upstream of the PIF promoter was predicted by AlphaFold Server(https://alphafold.com/) (accessed on 17 May 2024), which we further validated by Y1H (yeast one−hybrid) assay. Y1H assays were carried out to identify the specific binding of the candidate dofto the promoters of PIF. It was found that significant colony growth was observed in all groups on SD/− Ura/− Leu plates, indicating successful transformation and proper functioning of the system (Figure 10B). On the SD/−Ura/−Leu+100 AbA plate, the pPIF AbAi competent state transferred to the pGADT7 empty vector did not grow any colonies, while the p53 AbAi competent state transferred to the pGADT7Rec53 plasmid could grow larger colonies, indicating that the negative and positive controls worked normally in this system. The experimental group transferred with pGADT7 Dof plasmid was able to grow plaques on SD/−Ura/−Leu+100 AbA plates, indicating that PIF interacts with Dof.

## 3. Discussion

In previous studies, the PIF gene family played a crucial role in plant growth regulation, developmental processes, and response mechanisms to external environmental stimuli [41,42,43,44,45]. In soybean, *GmPIF4* had been identified to play an important biological function related to photoperiod and high temperature [46]. The overexpression of *GmPIF4* showed intact plant architecture and a faster transition from the flowering stage to the mature stage without yield losses [47]. In this study, a total of 22 *GmPIF* genes were screened. The similar physical and chemical properties of PIF proteins from various plant species support the hypothesis of their functional similarity [48,49]. The nucleus is where the PIF family’s transcription factors primarily operate. Evolutionary analyses of PIF proteins from soybean, Arabidopsis, maize, and rice were performed. The findings demonstrated that every PIF used in the comparison came from a common ancestor (Figure 1. The *GmPIF* genes within each subfamily had identical structures (Figure 2). Both GmPIF1d and GmPIF1c were clustered in class 2 and homologous with AtPIF1c, indicating that these PIF factors in the same evolutionary clade likely perform similar functions. It has been postulated that GmPIF1d and GmPIF1c perform the same function as AtPIF1 and are implicated in NAD(P)H dehydrogenase complex-mediated electron transfer for chlororespiration [50]. In contrast, GmPIF4c in class 4 was distantly related to other GmPIF. GmPIF7a and GmPIF7b were homologous to AtPIF7 and AtPIF8 in class 5. We speculated that GmPIF7a and GmPIF7b in class 5 may be involved in epigenetic reprogramming to promote transcriptional responses to shade in Arabidopsis [51]. GmPIF3h, GmPIF3b, and GmPIF3j were located in class 7, and GmPIF4a, GmPIF4b, and GmPIF4d were homologous to AtPIF4 and AtPIF5. PIF4 regulates microtubule organization and mediates high temperature-induced hypocotyl elongation in Arabidopsis [52,53,54]. GmPIF3k, GmPIF3g, and GmPIF3i were homologous to AtPIF3d. The SUMOylation of AtPIF3d leads to a decrease in AtPIF3d activity, which promotes cotyledon expansion, inhibits hypocotyl elongation under red light, and impairs PIF3-mediated gene induction and photoprotection [55]. GmPIF3a, GmPIF3c, GmPIF3d, GmPIF3f, and GmPIF3e clustered in class 12; GmPIF1a, GmPIF1b, and GmPIF1c were homologous to AtPIF1b clustered in class 13. By suppressing photosynthetic genes in the endodermis, AtPIF1b controls the development of plastids. An analysis of the motif and gene structure of the GmPIFs family members revealed that most of the GmPIFs members contained Motif1 (BHLH motif), and all members showed a similar exon–intron distribution pattern, which was similar to that of PIFs in other plant species [56,57,58]. The composition of the UTR and CDS of a gene is important for determining its function. The difference in the number of similar exons and introns within the same type of PIF indicates that the insertion event of introns has an impact on gene structure. This structural change may be a key factor driving functional differentiation among PIF members.

Protein interaction network analyses revealed 453 histone interactions in GmPIFs (Figure 5). GmPIFs can interact with each other as well as with the bHLH (H2EUM8_SOYBN) family of proteins. PIF3 regulates plant morphogenesis through its APB or APA structural domains, where it binds to PhyB or PhyA [59]. Respectively, PIFs play an important role in regulating the synthesis and signaling of GA by inducing the production of GA receptor GID1 and assembling it into GA-GID1 complexes. This complex binds to DELLA protein and promotes the ubiquitination degradation of the PIF protein, thereby inhibiting the activity of PIF in downstream gene transcription and affecting the growth and development process of plants [60,61]. In environments with insufficient light, key transcription factors PIF1, PIF3, and PIF4 in the light signaling pathway interact with upstream genes of FT (flowering time) and CO (CONSTANS) to form a CO-PIF complex that inhibits the binding of CO protein to the FT gene promoter, thereby preventing flowering. On the contrary, under sufficient light conditions, CO-PIF complexes do not form, and CO protein can promote the expression of FT genes, thereby driving the flowering process of plants [11]. These indicate that PIF3 protein plays an important role in the light, temperature, and hormone regulatory networks.

*GmPIF3g* is highly expressed in samples treated with different light temperatures and hormones. We can better explore the intricate network of soybean internode growth and plant height regulation because *GmPIF3g* is engaged in the intricate regulatory mechanisms of light temperature and hormones [44]. The pattern and degree of gene expression are significantly influenced by the promoter’s structure and sequence [41], following a more thorough investigation of the cis-regulatory elements of the GmPIF gene’s promoter region. According to the findings, the *GmPIF* gene’s response elements (Figure 7) closely match those observed in Arabidopsis. Both hormone-responsive and photo-responsive components are present in *GmPIF3g*. Previous studies have shown that PIF protein controls the ability of plants to tolerate high and low temperatures [48,62,63,64], and how *GmPIF3g* regulates gene networks under different light and hormone environments. We also investigated the *GmPIF3g* fishing protein. The promoter region is a key site for gene regulation, which contains specific sites for transcription factor recognition and binding. In this study, we predicted the core region of the *GmPIF3g* promoter at the MEME site and designed two probes, *GmPIF3g1* and *GmPIF3g2*, to further explore the interaction network of *GmPIF3g* by identifying proteins that interact with it through promoter probes. We developed a novel method using BLI technology, which offers advantages over the DNA pulldown technology, to identify promoter-interacting proteins [65]. This eliminates the time-consuming steps of isolating and purifying DNA–protein complexes, thereby reducing labor intensity. Additionally, it eliminates the need for extensive protein purification by directly capturing the peptide structure that binds to the DNA probes, thus saving time and resources. We discovered 29 key functional proteins that interact with *GmPIF3g* using this technique, such as the creation of cell walls, growth hormones, mitochondria, ubiquitination, and signal transmission, depend on these proteins (Table 3). These protein annotations suggest that proteins in several relevant regulatory pathways interact with the *GmPIF3g* promoter. By utilizing the Cell mPLOC database for subcellular localization prediction, we analyzed and found that out of the thirty identified proteins, seven were predicted to be located in the nucleus. This result suggests that these seven proteins play a major role in nuclear reactions. It has been previously demonstrated that light-activated photosensitive pigments are translocated to the nucleus to interact with PIF proteins, triggering the rapid degradation of PIF proteins [40,63,66]. The eight proteins were also found to be localized in the chloroplasts, and it has been shown that phy–PIF regulates chloroplast development by modulating light and genes. Moreover, PEP (plastid-encoded plastid RNA polymerase) activation in plastids during chloroplast biogenesis is redundantly repressed by multiple PIFs, and light-induced SIG (Small Inducible Gene) factors repressed by PIFs can act as cis-signals to activate PEPs in plastids and induce the expression of plastid photosynthesis genes. The molecular mechanism by which light and PIFs coordinate plastid transcription and nuclear photosynthesis through SIG cis-signaling has been revealed [67]. *FRS5*, a member of the FAR family, has been confirmed to be involved in the development of cell walls and can regulate the activity of the *GmPIF3g* promoter [68]. In addition, some transcription factors can bind DNA, RNA, or other proteins to participate in RNA metabolism, gene expression, or protein activity regulation [69,70,71,72].

The research found an interaction between PIF and Dof. We further validated the interaction between PIF and Dof using Y1H. Previous studies have shown that Dof transcription factors not only play an important role in regulating hormone signaling and responding to various biotic and abiotic stresses, but they are also widely involved in regulating various plant biological processes, such as carbon and nitrogen assimilation, dormancy, tissue differentiation, and carbohydrate metabolism [73,74,75,76]. The Arabidopsis DOF transcription factor COG1 in Arabidopsis regulates BR biosynthesis by binding to PIF4 and PIF5 promoters and inducing their expression, ultimately promoting hypocotyl growth. And AtCOG1 relies on light-induced photosensitizers but plays a negative regulatory role in the photosensitizer signaling pathway [77]. The overexpression of AtDOF 5.4/OBP4 leads to dwarfism in Arabidopsis plants by reducing cell size and quantity [78]. DOF protein can also enhance the survival ability of plants in extreme temperature environments. In Brassica plants, BnCDF1 is induced to express at low temperatures, and the overexpression of BnCDF1 enhances the plant’s cold tolerance [79]. DOF transcription factors have also been shown to mediate cell wall injury-induced wound healing and tissue regeneration [80]. All indicate that Dof is crucial in plant light-temperature and hormone signaling pathways. This is crucial for identifying upstream and downstream genes that interact with *GmPIF3g*, which will help us gain a deeper understanding of the regulatory network of *GmPIF3g* in light-temperature and hormone integration. Further research on the PIF regulatory network will help determine the roles of these downstream genes in plant development, growth, and environmental adaptation. It will also help identify novel breeding targets for plants. However, further studies are needed to determine how *GmPIF3g* specifically regulates these target genes and their functional changes under different physiological and environmental conditions. Among the fished proteins, we found an E3 ubiquitin ligase involved in syntaxin degradation, and PIF was modified by phytophosphorylation and degraded by ubiquitination in the light [81]; the precise alterations in this process are not entirely understood, though, and it is unclear if the pathway’s central element is an E3 ubiquitin ligase or a light-inducible protein kinase. These details should be clarified in the future.

## 4. Materials and Methods

### 4.1. Plant Materials and Growth Conditions

DN50 seeds provided by Northeast Agricultural University(Harbin, China) were sown in a nutrient bowl (diameter: 10 cm) and kept at 20–30 °C in a light incubator with a 16–8 h light–dark cycle. On the 9th day after germination, the seedlings grew faster throughout the entire elongation period, which is the time point characterized by a faster growth rate under shade conditions (500 μmol m^−2^s^−1^). To determine the effect of external hormones on the internode elongation of the soybean seedlings, 5 umol L^−1^ BR, 500 umol L^−1^ IAA, and 500 umol L^−1^ GA solutions were applied, and the epicotyls of the soybean seedlings were sampled 24 h later.

### 4.2. Physicochemical Property Analysis and Subcellular Localization Prediction of GmPIF

Drawing upon the eight PIF family members present in Arabidopsis. A total of 22 *GmPIFs* were successfully identified through a comparison with homologous protein sequences that are publicly available on the TAIR website (https://www.arabidopsis.org/) (accessed on 20 December 2022). The gene sequences and protein sequences of the *GmPIFs* were downloaded from the phytozome website (https://phytozome-next.jgi.doe.gov/) (accessed on 27 December 2022); the physicochemical properties of the soybean PIF proteins, including the number of amino acids, molecular weight, isoelectric point, and chromosomal location [82]. We used Expasy for analysis (http://web.expasy.org) (accessed on 6 January 2023), and the subcellular localization of the *GmPIF* protein was identified using the PSORT tool (https://psort.hgc.jp/) (accessed on 6 January 2023) [83].

### 4.3. Phylogenetic Tree Construction, Motif, and Gene Structure 

The amino acid sequences of soybeans (*GmPIFs*), maize (*ZmPIFs*), rice (*OsPIFs*), and *Arabidopsis thaliana* (*AtPIFs*) were analyzed using the MEGA11.0.13 software. Using the Arabidopsis PIF protein sequence as the template, the protein sequence databases of soybean, maize, and rice were searched to identify homologous proteins to PIF. Full-length sequences of all amino acids were selected, and multiple sequence comparisons were performed in the ClustalW program in the MEGA11.0.13 software. A phylogenetic tree was constructed using the neighbor-joining method, and the reliability of the node statistics in the tree was assessed by performing the bootstrap repetition test 1000 times [84]. Phylogenetic trees were plotted using the ChiPlot software (https://www.chiplot.online/) (accessed on 8 January 2023). The motif of the GmPIF protein was analyzed using MEME version 11.1.1 (http://meme-suite.org/) (accessed on 14 January 2023); the number of motif searches was 10, the length range was 6–100, and the rest of the parameters were defaults [85]. The GmPIF gene was extracted using the Gene Structure View program of the TBtools software (TBtools_windows–x64_1_098667, Jiangsu, China) to visualize the analysis results [86].

### 4.4. GmPIF Protein Secondary and Tertiary Structure Prediction

The secondary structure information of the GmPIF protein was obtained from the SOPMA (https://npsa-prabi.ibcp.fr/cgi-bin/npsa_automat.pl?page=npsa_sopma.html) (accessed on 28 January 2023) website [87]. The protein sequences of 22 soybean PIF genes were selected to construct tertiary structure models using the SWISSMODEL (https://swissmodel.expasy.org/) (accessed on 28 January 2023) [88].

### 4.5. Collinearity Relation of GmPIF

Based on the Ensemble Plants database (https://plants.ensembl.org/index.html) (accessed on 6 February 2023) [89], the DNA sequences, the GFF3 annotated file of soybean, and the linear relationships within soybean species were generated using the TBtools software.

### 4.6. GmPIF Protein Interaction Network Analysis

The STRING database (https://string-db.org/) (accessed on 8 April 2023)was selected to predict interaction network relationships with the *GmPIF* gene [30]. Protein and functional information were compared by referring to the UniProt (https://www.uniprot.org/) (accessed on 8 April 2023) and Soybase websites (https://www.soybase.org/) (accessed on 8 April 2023), respectively.

### 4.7. Quantitative Real-Time PCR Analysis

We extracted total RNA from soybean elongation internodes 24 h after hormone treatment. The quantitative detection of *GmPIF* gene expression levels after treatment with three exogenous hormones (IAA, GA, and BR) was performed using RT-PCR. The expression levels of *GmPIFs* from untreated elongating internodes were referred to as the controls (CK). The samples collected from 20 °C were referred to as LCK (no hormone treatment), LIAA (applied with 500 umol L^−1^ IAA solution), LGA (applied with 500 umol L^−1^ GA solution), and LBR (applied with 5 umol L^−1^ BR solution), while the samples collected from 30 °C were referred to as HCK (no hormone treatment), HIAA (applied with 500 umol L^−1^ IAA solution), HGA (applied with 500 umol L^−1^ GA solution), and HBR (Applied with 5 umol L^−1^ BR solution). Use RNA kit to extract RNA from the above samples (E.Z.N.A^®^ Plant RNA Kit, OMEGA, Beijing, China); use a spectrophotometer to check the concentration and integrity of RNA (≥100 ng/L), and calculate the required RNA dosage according to the kit (FSQ-301,TOYOBO, Beijing, China). After the thermal denaturation of RNA at 65 °C for 5 min, it was immediately cooled on ice. The reaction system is shown in Table 4. Reverse transcription to obtain soybean cDAN sequence. The reverse transcription reaction is shown in Table 5. All the primers used for the qRT–PCR analysis are listed in Supplementary Table S3 [90,91]. The preparation of real-time quantitative qRT-PCR (T100TM Thermal Cycler, Bio-Rad; USA) system, Table 6. The real-time quantitative qRT-PCR program settings are shown in Table 7. Each qRT–PCR reaction was performed thrice on three biological replicates (technical replicates), and the transcript levels of *GmPIF* were calculated based on the 2^−ΔΔCt^ method. The expression level of Actin 11 was used as an internal control to calculate gene expression. The data were analyzed and plotted using the TBtools and Excel software version 2010 [92].

### 4.8. Analysis of Cis-Acting Elements of the GmPIF Promoter

The promoter sequence up to 2000 bp upstream of the GmPIF gene was downloaded from the Phytozome database and analyzed using the PlantCARE software (http://bioinformatics.psb.ugent.be/webtools/plantcare/html/) (accessed on 30 April 2023) to predict the type and number of cis-acting elements in the promoter [93]. Visualization was performed using the TBtools software.

### 4.9. Promoter Probe Preparation of GmPIF3g

The 2000 bp sequence of the *GmPIF3g* promoter was downloaded from the Phytozome website. The promoter functional elements of the *GmPIF3g* gene were identified using the MEME (https://meme-suite.org/meme/) (accessed on 2 June 2023). A 100 bp sequence containing the key elements of the promoter was synthesized by Sangon Biotech (Shanghai, China). The single-stranded DNA with biotin-labeled 5′-end and 3′-end was synthesized by the Shanghai Bioengineering Company (Table 8), and the double-stranded DNA probes containing biotin at both ends were synthesized. The components of the annealing reaction system were as follows: 40 μL of nuclease-free water, 20 μL of annealing buffer for DNA oligos (5×), 20 μL of oligoA, and 20 μL of oligoB. The annealing reaction was performed in a gene-amplifying instrument (Veriti; Thermo Fisher Scientific, Waltham, MA, USA) with the following procedure: (1) The annealing reaction was performed at 95 °C for 2 min. (2) The reaction rate was decreased by 0.1 °C every 8 s to 25 °C, which took approximately 90 min.

### 4.10. Extraction of Total Soybean Protein

The total protein from the elongating internodes of the soybean samples was extracted using a cytosolic and cytoplasmic protein extraction kit (Beyoutime, Shanhai; China). Firstly, we took 60 mg of elongated internodes and cut them into small pieces. We mixed them with cytoplasmic protein extraction reagent AB [cytoplasmic protein reagent A (P0027–1) and cytoplasmic protein reagent B (P0027–2)] in a ratio of 20:1 (by volume). Afterwards, the tissues were homogenized on ice and transferred to a sterile centrifuge tube in an ice bath for 15 min; this step was repeated for another 15 min. Subsequently, the tissues were centrifuged at 15,000 rpm for 5 min at 4 °C, and the supernatant was aspirated to obtain cytoplasmic proteins. Ten volumes of cytosolic protein extract A1 [PMSF was added to cytosolic protein reagent A (P0027–1)] were added to the precipitate to reach the final concentration of 1 mmol/L. The tubes were shaken vigorously for 5 s and placed in an ice bath for 15 min; then, we added 10 μL of cytoplasmic protein extract B and kept it in an ice bath for 1 min. The solution was centrifuged at 13,000 rpm for 5 min at 4 °C after vortexing for 5 s. The cytoplasmic proteins were extracted by moving the supernatant to a fresh centrifuge tube. The final concentration of 1 mmol/L was then reached by adding 50 μL of cytosolic protein extract C [PMSF was added to cytosolic protein reagent (P0027–3)], and the supernatant was centrifuged at 13,000 rpm for 10 min at 4 °C. Finally, we transferred the supernatant cytoplasmic protein to a sterile centrifuge tube.

### 4.11. Fishing Proteins to Interact with GmPIF3

Fishing for proteins interacting with *GmPIF3* was conducted using a biolayer interferometry platform (Octet RED96; Pall, Port Washington, NY, USA). First, two activated streptavidin sensors with 200 μL of TE buffer were prepared. Then, two 200 μL (100 μg/mL) of biotin-labeled DNA, 400 μL of TE buffer, 200 μL of protein solution, 200 μL of protein solvent, and 200 μL of aqueous 0.1% formic acid by volume percent were prepared and added to the different wells of a 96-well plate in front-to-back order. Afterwards, the sensors were set to undergo baseline equilibration in TE buffer for 60 s. Subsequently, the sensors were immobilized in the wells and spiked with different DNA probes for 60 s; curing was completed after 60 s. The sensors were then fixed in the wells with different DNA probes. Then, the sensors with the immobilized probes as described above continued to undergo baseline equilibration in protein solvent for 60 s. Subsequently, the sensors with the immobilized probes were saturated bound (pendant) with 200 μL of the protein solution; after sufficient binding, the proteins bound to the different probe wells corresponding to the binding curves were eluted off separately using an elution solution (aqueous solution with a *v*/*v* ratio of 0.1% formic acid) to obtain the elution product, which was the protein containing the solution of the protein interacting with the promoter.

### 4.12. Mass Spectrometry Identification

Fishing proteins were identified using a high-resolution Q Exactive plus hybrid quadrupole-Orbitrap mass spectrometer (Thermo Fisher Scientific, Waltham, MA, USA). The identified peptide sequences were searched to match the proteins on the UniProt website (https://www.uniprot.org/) (accessed on 8 August 2023). The subcellular localization of the fished proteins was predicted using the cell–Ploc website (http://www.csbio.sjtu.edu.cn/bioinf/Cell-PLoc-2/) (accessed on 8 August 2023) [94].

### 4.13. Y1H

DNA binding sites were identified and analyzed by analyzing the expression of reporter genes in yeast cells [95]. Single colonies of p53-AbAi and pPIF-AbAi were picked and connected to YPDA liquid medium, respectively, and a small number of feelers were prepared. p53-AbAi feelers were single-transfected with pGADT7-Rec53 plasmid as a positive control. pPIF-AbAi feelers were single-transfected with pGADT7 and pGADT7-Dof plasmid, respectively, and the ones transfected with the empty pGADT7 were the negative control, and the latter was the experimental group. After the transformation was completed, the plasmids were coated on SD/-Ura/-Leu plates without AbA and SD/-Ura/-Leu plates with the lowest AbA inhibitory concentration and incubated at 30 °C for 4–5 days for observation. The six monoclones were picked from SD/-Ura/-Leu plates and added to 100 µL of 0.9% NaCl and mixed by shaking, and then spotted onto SD/-Ura/-Leu and the lowest AbA inhibitory concentration of SD/-Ura/-Leu plate, each with 5 µL of bacterial solution, incubated at 30 °C for 4–5 days (Boyuan Biological Company, Wuhan, China) [96].

## 5. Conclusions

In this study, we conducted bioinformatics analysis on the *GmPIF* gene family and investigated the effects of GA, BR, and IAA hormone treatments on changes in *GmPIF* gene expression under different environmental conditions. Our results revealed that *GmPIF3* was significantly expressed, prompting us to develop a new method to identify the reciprocal proteins of *GmPIF3.* This allowed us to establish a network of Dof-GmPIF3-regulated genes. While our findings provide valuable insights into the molecular function of *GmPIF3*, further research is necessary to gain a more comprehensive understanding of its role in plant growth and development.

## Figures and Tables

**Table 1:** Physicochemical properties and subcellular localization of the *GmPIF* gene family.

Name	Gene ID	Number of Amino Acids (aa)	Molecular Weight (kD)	Isoelectric Point (pI)	Chromosome Number Location	Instability Index	Subcellular Localization
GmPIF1a	Glyma.03G170300	502	55.04	5.86	Chr03	67.51	Nuclear
GmPIF1b	Glyma.10G042800	489	54.02	5.84	Chr10	65.8	Nuclear
GmPIF1c	Glyma.13G130100	509	56.36	5.68	Chr13	62.48	Nuclear
GmPIF1d	Glyma.07G029900	272	29.76	8.05	Chr07	34.14	Nuclear
GmPIF1e	Glyma.08G213000	277	30.32	6.44	Chr08	44.53	Nuclear
GmPIF3a	Glyma.01G187600	375	40.90	5.61	Chr01	76.09	Nuclear
GmPIF3b	Glyma.03G225000	517	57.39	8.75	Chr03	57.03	Nuclear
GmPIF3c	Glyma.11G054600	381	41.50	5.87	Chr11	73.06	Nuclear
GmPIF3d	Glyma.14G084200	297	32.63	8.5	Chr14	68.25	Nuclear
GmPIF3e	Glyma.17G180800	296	32.40	6.4	Chr17	68.22	Nuclear
GmPIF3f	Glyma.17G241000	160	18.48	9.16	Chr18	89.58	Nuclear
GmPIF3g	Glyma.10G142600	691	74.15	6.43	Chr10	59.7	Nuclear
GmPIF3h	Glyma.10G138800	449	49.16	8.33	Chr10	51.75	Nuclear
GmPIF3i	Glyma.19G224700	633	68.91	5.72	Chr19	53.17	Nuclear
GmPIF3j	Glyma.19G222000	395	43.83	9.37	Chr19	51.5	Nuclear
GmPIF3k	Glyma.20G091200	722	77.25	5.95	Chr20	58.88	Nuclear
GmPIF4a	Glyma.02G282100	562	62.23	6.58	Chr02	59.27	Nuclear
GmPIF4b	Glyma.08G303900	525	57.81	6.41	Chr08	54.57	Nuclear
GmPIF4c	Glyma.14G102200	562	62.11	6.97	Chr14	55.43	Nuclear
GmPIF4d	Glyma.18G115700	547	60.53	6.58	Chr18	61.23	Nuclear
GmPIF7a	Glyma.01G076900	458	49.17	8.88	Chr01	47.61	Nuclear
GmPIF7b	Glyma.03G034000	397	44.19	9.41	Chr03	51.91	Nuclear

**Table 2:** Secondary structure and subcellular localization of *GmPIFs*.

Name	α-Helix	β-Sheet	Random Coil	Extended Strand
GmPIF1a	27.60%	1.99%	66.14%	4.18%
GmPIF1b	29.94%	2.24%	62.93%	4.89%
GmPIF1c	26.89%	2.52%	64.71%	5.88%
GmPIF1d	15.07%	8.64%	51.84%	24.63%
GmPIF1e	18.05%	8.66%	48.01%	25.27%
GmPIF3a	24.00%	1.33%	65.07%	9.60%
GmPIF3b	21.47%	2.13%	69.05%	7.35%
GmPIF3c	25.20%	2.36%	60.10%	12.34%
GmPIF3d	23.23%	2.36%	62.63%	11.78%
GmPIF3e	32.50%	3.12%	52.50%	11.88%
GmPIF3f	27.36%	2.70%	60.14%	9.80%
GmPIF3g	17.80%	2.17%	72.65%	7.38%
GmPIF3h	26.50%	1.11%	65.70%	6.68%
GmPIF3i	21.01%	1.90%	69.98%	7.11%
GmPIF3j	28.19%	2.90%	60.04%	8.88%
GmPIF3k	19.67%	2.35%	71.88%	6.09%
GmPIF4a	21.53%	2.85%	69.40%	6.23%
GmPIF4b	23.24%	2.10%	68.95%	5.71%
GmPIF4c	21.71%	2.14%	70.82%	5.34%
GmPIF4d	21.94%	1.83%	71.12%	5.12%
GmPIF7a	27.51%	1.09%	67.03%	4.37%
GmPIF7b	33.50%	1.51%	59.95%	5.04%

**Table 3:** Main functional proteins regulating the *GmPIF3g* promoter.

DNA	Protein	Description	Subcellular Localization
*GmPIF3g1*	A0A178UH44	Signal recognition particle, endoplasmic reticulum targeting	Cytoplasm
*GmPIF3g1*	A0A654FBE6	Small GTPase-mediated signal transduction	Golgi apparatus, nucleus
*GmPIF3g1*	I1LHH9	Dof-type zinc finger DNA-binding family protein	Nucleus
*GmPIF3g1*	I1M744	Response to auxin stimulus	Nucleus
*GmPIF3g1*	K7L9W3	Mitochondrion	Cytoplasm
*GmPIF3g1*	K7LKV8	Cellulose synthase (UDP-forming)	Chloroplast, Golgi apparatus
*GmPIF3g1*	K7LWI4	Mitochondrion	Chloroplast
*GmPIF3g1*	Q0WLB4	Floral repressor gene FLOWERING LOCUS C (FLC)	Chloroplast nucleus
*GmPIF3g2*	A0A0R0JEC6	Steroid biosynthetic process	Chloroplast, Golgi apparatus
*GmPIF3g2*	Q9ZPT6	FRS5; regulation of transcription, DNA-templated	Chloroplast
*GmPIF3g2*	A0A0R0JTV5	Translation elongation factor EF–1 alpha/Tu	Cytoplasm, nucleus
*GmPIF3g2*	A0A0R0KHR2	Carbohydrate metabolic process	Cell membrane
*GmPIF3g2*	A0A178U7H3	Folic acid-containing compound metabolic process	Peroxisome
*GmPIF3g2*	A0A1P8ATW4	Golgi organization	Cell membrane, nucleus
*GmPIF3g2*	A0A1P8B118	Raumatin and (Z)–3–hexen–1–yl acetate biosynthesis	Nucleus
*GmPIF3g2*	A0A654G701	E3 ubiquitin ligase involved in syntaxin degradation	Nucleus
*GmPIF3g2*	A0A7G2E5T9	Negative regulation of endopeptidase activity	Vacuole
*GmPIF3g2*	A0A7G2E6Q4	Folic acid-containing compound biosynthetic process	Chloroplast
*GmPIF3g2*	A0A7G2E7M8	Protein acetyltransferase complex	Nucleus
*GmPIF3g2*	A0A7G2F610	Ubiquitin activating enzyme 2	Nucleus
*GmPIF3g2*	C6TLV3	Mitochondrial respiratory chain complex IV assembly	Chloroplast peroxisome
*GmPIF3g2*	I1JKS8	Secondary metabolite biosynthetic process	Endoplasmic reticulum
*GmPIF3g2*	I1LL69	Cell wall	Cell wall
*GmPIF3g2*	I1MMT5	Tonoplast monosaccharide transporter3	Cell membrane
*GmPIF3g2*	K7KQ57	Superpathway of acetyl–CoA biosynthesis	Chloroplast
*GmPIF3g2*	K7L045	Signal transduction	Cell membrane
*GmPIF3g2*	K7L7Q1	Indole–3–acetate activation I	Endoplasmic reticulum
*GmPIF3g2*	K7LBX5	Signal transduction	Nucleus
*GmPIF3g2*	K7LWI4	Chloroplast plastid thylakoid	Chloroplast
*GmPIF3g2*	Q5GFS0	Mitochondrion	Chloroplast, mitochondrion

**Table 4:** RNA reaction system.

Components	The Amount Every Tube (µL)
5x RT Master Mix RNA template	2 µL 1 pg^−1^ μg
Nuclease-free water	To 10 µL

**Table 5:** Reverse transcription program.

Temperature	Time		
37 °C 15 °C	15 min 5 min	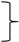	Reverse transcription reaction
98 °C	10 s		Enzyme inactivation
4 °C	∞ (preserve)		

Note: After the reaction, store at −20 °C. Real-time PCR, as a template directly or after dilution.

**Table 6:** qRT-PCR system.

Components	The Amount Every Tube (µL)
GREEN Master Mix	10
Primer1 (10 µM)	0.5
Primer2 (10 µM)	0.5
cDNA template	1
ddH_2_O	8

**Table 7:** qRT-PCR program.

Program	Cycle	Temperature	Time
Stage 1 Pre-denaturation	Reps: 1	95 °C	5 min
Stage 2 Cyclic reaction	Reps: 40	95 °C	10 s
		55 °C	30 s
Stage 3 Dissolution curve		Machine self-contained	

**Table 8:** Key promoter sequence of *GmPIF3g*.

Primer Name	Primer Sequence
*GmPIF3g1–DNA oligoA*	5′TTGAGTTGACCCCACCAACACAACACACACTTAAGGACTTACGACGACTAATCCCTTTTTGTTTTTTCTTTCTTTCTTTTCTTATTTTTATTCTAACTTA3′
*GmPIF3g1–DNA oligoB*	3′AACTCAACTGGGGTGGTTGTGTTGTGTGTGCCAACCTGAATGCTGCTGATTAGGGAAAAACAAAAAAGAAAGAAAGAAAAGAATAAAAATAAGATTGAAT5′
*GmPIF3g2–DNA oligoA*	5′CTTAAACCACGACAACTTTTGACTAAACCATGGTTAGAACTTAGAAAGTAGAAACCCCTGAATTTCTCACTCTTTTCTGTTCCTGTCTGTCTCTTTTGTG3′
*GmPIF3g2–DNA oligoB*	3′GAATTTGGTGCTGTTGAAAACTGATTTGGTACCAATCTTGAATCTTTCATCTTTGGGGACTTAAAGAGTGAGAAAAGACAAGGACAGACAGAGAAAACAC5′

## Data Availability

Data are not available due to privacy or moral restrictions.

## Supplementary Materials

The following supporting information can be downloaded at: https://www.mdpi.com/article/10.3390/ijms26020551/s1.
